# The analysis and control of scale accumulation for mixed layer injection of water for the Shuanghe oil area in Yanchang Oilfield

**DOI:** 10.1038/s41598-026-47479-6

**Published:** 2026-04-09

**Authors:** Chunmin Qi, Yu Xia, Shanfa Tang

**Affiliations:** 1 School of Petroleum Engineering, Yangtze University, Wuhan, 430100 China; 2 Hubei Key Laboratory of Oil and Gas Drilling and Production Engineering, Yangtze University, Wuhan, 430100 Hubei Province China; 3 School of Petroleum Engineering, National Engineering Research Center for Oil & Gas Drilling and Completion Technology, Yangtze University, Wuhan, 430100 China; 4 State Key Laboratory of Low Carbon Catalysis and Carbon Dioxide Utilization(Yangtze University), Wuhan, 430100 China

**Keywords:** Waterflooding, Scale tendency, Mechanism, Process scale inhibition, Shuanghe oil area, Engineering, Environmental sciences, Hydrology

## Abstract

Waterflooding remains one of the primary techniques for developing low-permeability reservoirs. However, scale accumulation caused by incompatible water mixing can lead to pore blockage and formation damage, resulting in elevated injection pressure and reduced water injection efficiency, which ultimately undermines the effectiveness of such development efforts. This study focuses on the Shuanghe oil area within the Yanchang Oilfield, China, where three distinct water sources—produced water from the Chang 6 reservoir, produced water from the Yan’an Formation, and surface water—are utilized for injection purposes. To address the challenges associated with water injection in this block, we conducted a comprehensive set of investigations, including reservoir sensitivity experiments, injected water quality analysis, and compatibility studies; notably, the wastewater treatment process has been optimized. Through these efforts, we systematically examined the conditions that lead to the formation of plugging factors during water injection. Our findings reveal that poor compatibility between the different injected water sources readily triggers the precipitation of inorganic scale upon mixing, which in turn clogs reservoir pores and drives up injection pressure. Specifically, when water samples from different sources in the Shuanghe oil area are mixed, they precipitate three main types of scale: calcium carbonate (CaCO₃), barium sulfate (BaSO₄), and strontium sulfate (SrSO₄). Among these, calcium carbonate and barium sulfate were the most abundant in mixtures of produced waters. Additionally, we observed a significant increase in calcium carbonate scale formation as both temperature and pH levels rose. Targeting the specific scale types identified in the block’s mixed-layer water, we implemented a two-pronged control strategy: pre-mixing the effluent waters and adding pH regulators. This approach proved effective in suppressing the formation of both carbonate and sulfate scales. Finally, core experiments were performed to assess the extent of reservoir damage caused by injected water. The results showed that after treatment, the core damage rate associated with mixed water injection was reduced to 15%.

**Supplementary Information:**

The online version contains supplementary material available at 10.1038/s41598-026-47479-6.

## Introduction

Located on China’s Loess Plateau, the Yanchang Oilfield is a typical representative of low-permeability and ultra-low-permeability reservoirs, with many of its oil-bearing blocks having entered the middle or late stages of development^1,2^. To sustain reservoir pressure and boost oil recovery, secondary oil recovery technologies have been widely applied across the Yanchang Oilfield. Among these, the most common and cost-effective approach involves injecting large volumes of water into oil reservoirs to offset the pressure drop caused by oil extraction^3,4^.

During oil and gas production, substantial volumes of wastewater—commonly termed “produced water”—are generated. However, due to regional water scarcity and environmental protection requirements, this produced water must undergo pre-treatment before being reinjected back into the reservoir^5,6^.

As is widely recognized, scale deposition poses a severe challenge to water injection systems, particularly when two incompatible fluids are mixed. Inorganic scales form as a result of variations in temperature, pressure, pH, and other factors related to the incompatible ionic composition of the injected water^7–9^. In waterflood development, produced water—typically characterized by high salt content, suspended solids, and bacteria—is often used as injection water to enhance oil recovery^10^. Unfortunately, low-quality injected water can lead to scale deposition and clogging of injection pipelines and formation pores, disrupting normal oil production. Moreover, long-term use of such mixed water causes progressive pore throat blockage, which reduces reservoir injectivity. To maintain target injection rates, the injection pressure must be increased; this gradual pressure rise is a key indicator of formation damage and ultimately leads to reduced water injection volumes and decreased production from associated oil wells. To date, numerous studies have addressed the compatibility of injected water, covering aspects such as laboratory-based compatibility testing^11–13^, theoretical prediction of scaling tendencies, and on-site adjustments to treatment processes.

To mitigate scaling, various technologies have been developed, which can be broadly categorized into chemical, physical, and process-based methods. Chemical inhibition, typically involving the continuous injection of scale inhibitors (e.g., phosphonates, polymers), is the most widely used approach due to its immediate effectiveness. However, its long-term application raises concerns about escalating operational costs, potential formation damage, and environmental impact from chemical accumulation. Physical methods, such as magnetic or ultrasonic treatment, offer chemical-free alternatives by aiming to alter crystallization dynamics. Nevertheless, their efficacy in complex, large-scale oilfield environments is often inconsistent and heavily dependent on specific water chemistry and flow conditions^14,15^. In contrast, process-based inhibition focuses on preventing scale formation by modifying the injection water itself before it enters the reservoir—for instance, through ion removal (e.g., sulfate reduction) or controlled mixing to precipitate scaling ions at surface facilities^16–18^. This strategy addresses the root cause of incompatibility and can provide a more fundamental and potentially sustainable solution, particularly for scaling dominated by specific, high-concentration incompatible ion pairs^19–21^.

Furthermore, the spatial distribution and severity of scale deposition can be significantly influenced by reservoir heterogeneity and rock wettability. Variations in pore structure and mineral surface properties can create preferential flow paths and localized sites for ion accumulation and precipitation, thereby exacerbating scaling damage. Similar coupled effects of geochemistry and reservoir properties on fluid-rock interactions have been extensively studied in the context of CO₂-brine systems and geological carbon storage^22–24^.

In the Shuanghe oil area of the Yanchang Oilfield, three types of water sources are used for injection into the Chang 6 reservoir: produced water from the Chang 6 reservoir itself, produced water from the Yan 9 and Yan 10 reservoirs, and surface water. The produced water from the Chang 6 reservoir is characterized by high mineralization, with notably high concentrations of calcium (Ca²⁺), strontium (Sr²⁺), and barium (Ba²⁺) ions. Per Sulin’s water classification system, this water falls into the calcium chloride (CaCl₂) type. In contrast, the produced water from the Yan 9 and Yan 10 reservoirs is rich in anions such as bicarbonate (HCO₃⁻), carbonate (CO₃²⁻), and sulfate (SO₄²⁻), while its calcium ion content remains low^25–27^. This gives it the properties of a sodium bicarbonate (NaHCO₃) water type, and it exhibits alkaline characteristics. As for the surface water, its calcium content is higher than that of the Yan’an Formation produced water, but it contains lower levels of HCO₃⁻, SO₄²⁻, and CO₃²⁻, resulting in a neutral pH.

In the Shuanghe oil area’s current field operation, all three water sources—produced water from the Chang 6 reservoir, produced water from the Yan’an Formation, and shallow groundwater from the Luohe aquifer—are collected together in the central wastewater treatment station, treated jointly, and then reinjected into the Chang 6 reservoir for pressure maintenance. This operational necessity, driven by logistical and economic considerations, inevitably leads to mixing of these incompatible waters. Therefore, our study aims to diagnose the scaling problems arising from this existing operational practice and develop targeted mitigation strategies.

**Fig. 1:**
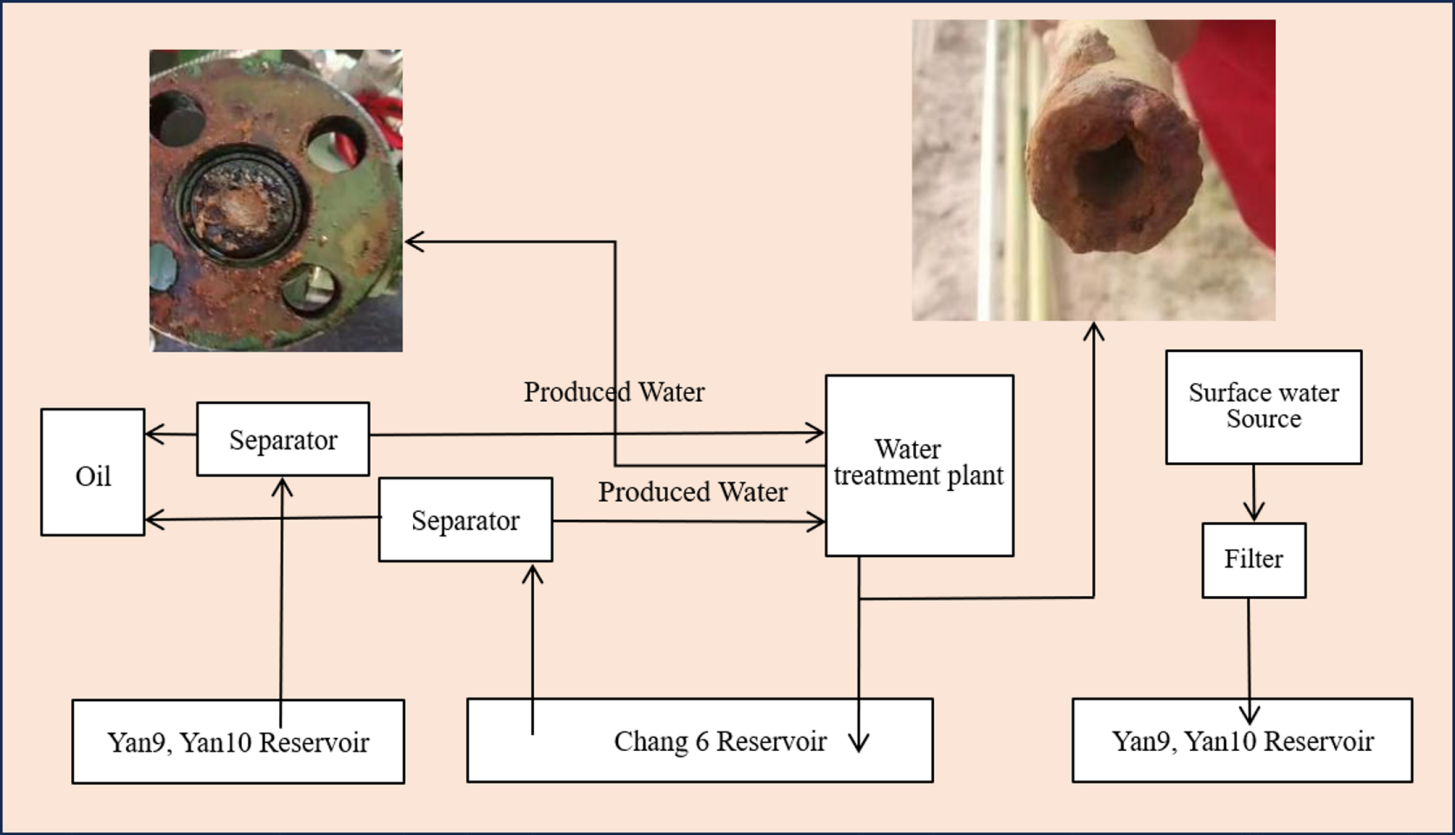
The photos of the pipeline scaling.

Due to their distinct compositional traits, these three water sources exhibit poor compatibility. When they interact, intense chemical reactions occur, leading to scale formation. Such scale can accumulate in injection equipment, pipelines, and wellbores, causing blockages. Even more problematic, if these chemical reactions take place within the reservoir, the resulting scale will clog the formation’s pore spaces, reducing its permeability^28–30^. Both scenarios severely hinder oil production rates and undermine the overall efficiency of waterflood recovery. Figure 1 shows the condition of water injection pipelines in the Shuanghe block, clearly revealing substantial scale deposits.

Furthermore, the spatial distribution and severity of scale deposition can be significantly influenced by reservoir heterogeneity and rock wettability. Wettability affects scale nucleation and growth: water-wet surfaces provide favorable sites for crystal attachment, while oil-wet surfaces tend to confine precipitation to the pore interior water phase. Wettability contrasts create preferential flow paths where ions can accumulate and precipitate. This understanding, while not directly actionable for reservoir-scale wettability modification, helps predict high-risk zones for scale deposition and guides the strategic placement of chemical inhibitors or the design of water injection patterns.

The objective of this study is to investigate the compatibility of three water sources—produced water from the Chang 6 reservoir, produced water from the Yan 9 and Yan 10 reservoirs, and surface water—while utilizing the ScaleChem software to predict the scaling potential under different mixing ratios. Additionally, the study evaluates the sensitivity of the reservoir to water, salt, and flow rate. By leveraging theoretical predictive models, the critical factors driving scale formation during water injection are identified. To address scaling issues, the study integrates process optimization with the existing wastewater treatment workflow: two key measures are implemented—first, pre-mixing the two types of produced water to trigger a chemical reaction that generates sulfate scale (thereby removing sulfate ions from the mixed water), and second, adding pH regulators to adjust the pH of the injected water to 6.0–6.5 (matching the pH of the Chang 6 reservoir’s in-situ water). These steps ensure the reinjected water is compatible with the Chang 6 reservoir water. Finally, core damage experiments are conducted to gain deeper insights into how water injection affects reservoir performance.

## Experimental part

### Instruments and reagents

Tables 1 and 2 lists the main instruments and reagents used in this study, along with their specifications and sources.

**Table 1:** Main instruments and reagents.

Instrument	Model	Manufacturer
ICP-OES	Optima 2100DV	PerkinElmer
pH meter	pHS-25	Shanghai Yi Electrical Scientific Instrument Co., Ltd.
Electronic analytical balance	BSA124S-CW	Sartorius
Core high temperature and high pressure displacement test device	LDY-IV	Haian County Petroleum Scientific Research Instrument Co., Ltd.
X-ray diffractometer (XRD)	D8 advance	Bruker

**Table 2:** Main reagents.

Reagent	Purity/Grade	Supplier
Disodium EDTA (Ethylene diamine tetraacetic acid)	AR	Tianjin Fengchuan Chemical Reagent Technology Co., Ltd.
Barium chromate	AR	Tianjin Kemiou Chemical Reagent Co., Ltd.
Silver nitrate	AR	Guangzhou Jinhua Chemical Reagent Co., Ltd.
Ammonium chloride	AR	Tianjin Beilian Fine Chemicals Development Co., Ltd.
Ammonia water	AR	Tianjin Beilian Fine Chemicals Development Co., Ltd.
Hydrochloric acid	AR	Kaifeng Zhongping Chemical Group Co., Ltd. Reagent Factory
Scale inhibitor (hydroxy ethylenediphosphonic acid)		Fengyuan Company of Yanchang Oilfield

### Experimental methods

#### Analysis of ionic composition of water samples

The water samples were sourced from the produced water of the Yan’an Formation and the Chang 6 reservoir, respectively. The surface water was collected from the Luohe layer at a depth of 300 m. Its calcium content (127.5 mg/L) is higher than that of the Yan’an Formation produced water (26.13 mg/L), which is attributed to the dissolution of calcium-bearing minerals in the shallow aquifer. After filtering the water samples through a 0.45 μm filter membrane, the compositional analysis of the water samples was conducted in accordance with the oil and gas industry standard SY/T 5523 − 2016 “Oilfield Water Analysis Method” of the People’s Republic of China.

#### Scaling trend prediction

The ScaleChem3.0 scale prediction software was utilized for the prediction study. The measured ion concentrations in the solution were input into the corresponding prediction formulas for calculation.

#### Core flow test

**Fig. 2:**
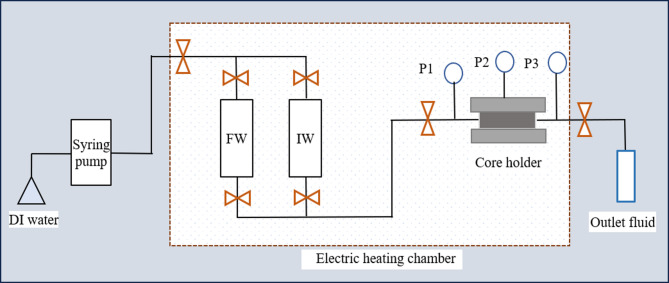
Flow-process diagram of core flow test; P1, injection pressure gauge; P2, confining pressure gauge; P3, outflow pressure gauge.

Core flow experiments were conducted in accordance with the oil and gas industry standard SY/T 5358 − 2010 “Formation Damage Evaluation by Flow Test”. Cores from the Chang 6 reservoir of the oil area were selected, with diameters and lengths of 25.4 mm and 60.0 mm, respectively. The cores were dried in an oven at 100 °C for 24 h, then vacuumed, and their weight was measured. Their weight was measured again after the cores were completely saturated with formation water. For each core, the pore volume was calculated based on the difference between the saturated and dry weights, and the porosity was determined by the ratio of the pore volume to the total volume of the core. To determine the core permeability, formation water was injected at a flow rate of 0.2 mL/min, the pressure difference across the core was recorded, and the initial permeability was calculated using Darcy’s law. The flow-process diagram of core flow test was shown in Fig. 2.

After establishing the initial porosity and permeability of the cores, core sensitivity tests were conducted. The injection water (both non-optimized and optimized) was injected into each core, the flow pressure drop across 10 PV (Pore volume) was measured, and variations in core permeability were calculated to then determine the core damage rate. The core damage rate was defined based on permeability reduction and calculated using the following formula:

1 $$\:\mathrm{D}\mathrm{a}\mathrm{m}\mathrm{a}\mathrm{g}\mathrm{e}\:\mathrm{R}\mathrm{a}\mathrm{t}\mathrm{e}\:\left(\mathrm{\%}\right)=\frac{{K}_{\mathrm{i}\mathrm{n}\mathrm{i}\mathrm{t}\mathrm{i}\mathrm{a}\mathrm{l}}-{K}_{\mathrm{f}\mathrm{i}\mathrm{n}\mathrm{a}\mathrm{l}}}{{K}_{\mathrm{i}\mathrm{n}\mathrm{i}\mathrm{t}\mathrm{i}\mathrm{a}\mathrm{l}}}\times\:100\%$$

Where *K*_initial_ is the initial core permeability measured with formation water, and *K*_final_ is the permeability after injecting 10 pore volumes of the test water. Permeability was used as the primary indicator because it is more sensitive to pore-throat blockage caused by scale precipitation than porosity.

All dynamic experiments were conducted at a reservoir temperature of 50 °C. Key experiments, including core flow tests and ion concentration measurements, were performed in triplicate to ensure reproducibility. The average values are reported, with relative standard deviations less than 5% for permeability measurements and less than 2% for ICP-OES analyses.

## Results and discussion

### Basic characteristics of reservoir and injected water

#### Reservoir sensitivity evaluation

Reservoir sensitivity is a well-documented factor that often leads to increased water injection pressure and reservoir damage23. For this study, the Chang 6 reservoir— the primary production reservoir in the Shuanghe Oilfield—was selected as the experimental subject. In accordance with standard reservoir sensitivity test protocols, quantitative evaluations were conducted to assess the water sensitivity, velocity sensitivity, and salt sensitivity of the Chang 6 reservoir. The results of these evaluations are summarized in Table 3.

Reservoir water sensitivity is closely associated with the type and content of clay minerals present, which is primarily governed by water-sensitive minerals such as montmorillonite and illite-smectite mixed-layer clay24. Experimental studies on clay-rich formations have further demonstrated how mineral composition and associated wettability can dictate patterns of ionic precipitation and subsequent fluid migration. In the case of the Chang 6 reservoir, water sensitivity-induced damage was determined to be weak; this is attributed to the low total content of clay minerals in the reservoir formation.

For salt sensitivity, test results showed that when the salinity of the injected water exceeds 500 mg/L, the average reservoir damage rate is 15.2%. This level of damage is insufficient to trigger clay swelling in the reservoir or cause significant formation impairment^31–33^.

In the velocity sensitivity tests, core permeability decreased gradually as the flow rate increased. Notably, there was no obvious critical flow rate at which permeability dropped sharply by 5%—a key threshold for defining significant velocity sensitivity. This observation indicates that the Chang 6 reservoir exhibits generally weak sensitivity to flow velocity.

Collectively, these experimental results confirm that the production reservoir (Chang 6 reservoir) of the Shuanghe Oilfield has weak velocity sensitivity, weak water sensitivity, and weak salt sensitivity. Thus, reservoir sensitivity is not the primary driver of high water injection pressure and reservoir damage in this oilfield. It is noteworthy that while the intrinsic mineralogical sensitivity of the Chang 6 reservoir is weak, the subsequent precipitation of inorganic scales within the pore network can itself alter local wettability and create additional flow barriers. This secondary effect, where precipitation modifies fluid-rock interactions and multiphase flow properties, has been observed in studies of CO₂-brine systems and clay-rich formations. It is important to distinguish between reservoir sensitivity and scale precipitation. Reservoir sensitivity refers to the intrinsic response of reservoir minerals (particularly clays) to foreign fluids—weak water/salt sensitivity means the reservoir’s clay minerals have low swelling/dispersion potential. Scale precipitation, in contrast, results from chemical reactions between dissolved ions in different water sources, forming new solid phases (CaCO₃, BaSO₄, SrSO₄) that are not originally present in the reservoir. Even a reservoir with weak intrinsic sensitivity can suffer severe permeability damage from scale plugging because scale particles are introduced into pore throats from the injected water. Therefore, this study addresses the scaling problem arising from fluid-fluid incompatibility, independent of the reservoir’s intrinsic mineral sensitivity.

From a practical perspective, understanding the role of wettability in scale deposition can inform mitigation strategies. Although reservoir-scale wettability modification is challenging, knowledge of wettability distribution helps identify zones most susceptible to scaling (e.g., water-wet regions or wettability boundaries). This information can guide the targeted placement of scale inhibitors or the optimization of water injection schemes to minimize scale-related formation damage.

**Table 3:** The results of reservoir sensitivity evaluation.

Item	Core number	Damage rate %	Sensitive index %	Level of sensitivity
Velocity sensitivity	Shuang 65-s1	7.0	10.4	Weak velocity sensitivity
Water sensitivity	Shuang 68 − 11	13.3	23.5	Weak Water sensitivity
Salt sensitivity	Shuang 67-s13	5.3	–	Weak Salt sensitivity

#### Ion composition analysis of injected water

Accurate analysis of ion content in injected water is critical, as it serves as the foundational data for all subsequent research. Conventional ion concentrations in the injected water were determined following the standard method detailed in Sect.  1.2, while the concentrations of strontium ions (Sr²⁺) and barium ions (Ba²⁺) were specifically measured using inductively coupled plasma optical emission spectrometry (ICP-OES). The analysis results, summarized in Table 4, confirm that the produced water in the Shuanghe Oilfield consists of two distinct water types: sodium bicarbonate (NaHCO₃) water from the Yan’an Formation and calcium chloride (CaCl₂) water from the Yanchang Formation (Chang 6 reservoir).

The produced water from the Yan’an Formation is characterized by low concentrations of magnesium ions (Mg²⁺) and calcium ions (Ca²⁺) (both < 50 mg/L), with no detectable Sr²⁺ or Ba²⁺. Its average concentrations of chloride ions (Cl⁻), bicarbonate ions (HCO₃⁻), carbonate ions (CO₃²⁻), and sulfate ions (SO₄²⁻) are 4590 mg/L, 2076 mg/L, 789.0 mg/L, and 1188 mg/L, respectively. This water exhibits a distinct alkaline property and is classified as the NaHCO₃ water type.

In contrast, the formation water from the Chang 6 reservoir has high salinity, with an average total dissolved solids (TDS) content of 52,880 mg/L. It contains notably high concentrations of Ca²⁺, Sr²⁺, and Ba²⁺, with average values of 2434 mg/L, 408.0 mg/L, and 225.9 mg/L, respectively. Additionally, this water has a high Cl⁻ concentration and a pH range of 6.0–6.5, consistent with the CaCl₂ water type classification.

The surface water from the Luohe layer is characterized by low concentrations of scale-forming anions, including HCO₃⁻, CO₃²⁻, and SO₄²⁻. However, its compatibility with the aforementioned formation waters (from the Yan’an Formation and Chang 6 reservoir) requires further evaluation—this is because any incompatibility could directly impact scale formation risks and subsequent wastewater treatment process design^34^.

**Table 4:** Analysis results of ion composition of injected water.

Item	Yan ‘an formation produced water mg/L	Chang 6 reservoir produced water mg/L	Suface water mg/L
Na^+^+K^+^	5505	16,837	66.78
Mg^2+^	8.72	619.3	2.80
Ca^2+^	26.13	2434	127.5
Sr^2+^	0	408.0	0
Ba^2+^	0	225.9	0
HCO_3_^−^	2076	139.2	280.6
CO_3_^2−^	789.0	0	39.43
Cl^−^	4590	32,198	55.27
SO_4_^2−^	1188	19.0	34.0
pH	8.5	6.2	6.9
Salinity	14,183	52,880	606.4
Water type	NaHCO_3_	CaCl_2_	NaHCO_3_

### Scaling tendency and compatibility of injected water

#### Scaling trend prediction

Waterflooding has become one of the most effective techniques for enhancing oil recovery in oilfield operations. However, inorganic salt scaling during water injection remains a significant and unavoidable challenge. To develop targeted anti-scaling and descaling strategies, it is critical to scientifically and accurately predict the scaling tendency of injected water in oilfields.

ScaleChem—a scale prediction software co-developed by OLI Systems and Shell—can predict scaling for up to 50 different minerals25. Its capabilities cover scaling reactions that may occur in oil and gas fields globally, as well as in industrial equipment. By leveraging its extensive built-in database, ScaleChem can simulate reservoir conditions using water composition analysis data, and predict key parameters such as potential (or already formed) scale types, scaling trends, and scale quantities. The thermodynamic basis of scaling prediction, which considers the coupling of ion concentrations, temperature, pressure, and pH to calculate mineral saturation, is conceptually similar to the framework used in reactive transport models for CO₂ storage. These models also emphasize the critical interplay between salinity, pH, and mineral stability in governing long-term geochemical evolution^35^.

In this study, OLI ScaleChem 3.0 was employed as the core prediction tool to investigate the scaling behavior of two sets of water mixtures: (1) mixtures of produced water and surface water, and (2) mixtures of produced waters from different strata. All simulations were conducted under conditions mimicking the actual reservoir environment, specifically a temperature of 50 °C, a pressure of 10 MPa, and a pH of 7. Through analysis of the predicted data, the study successfully identified how mixing ratios affect scale formation trends—providing valuable insights for addressing scaling issues in waterflood development projects.

#### Compatibility of Yan’an formation produced water and surface water

**Fig. 3:**
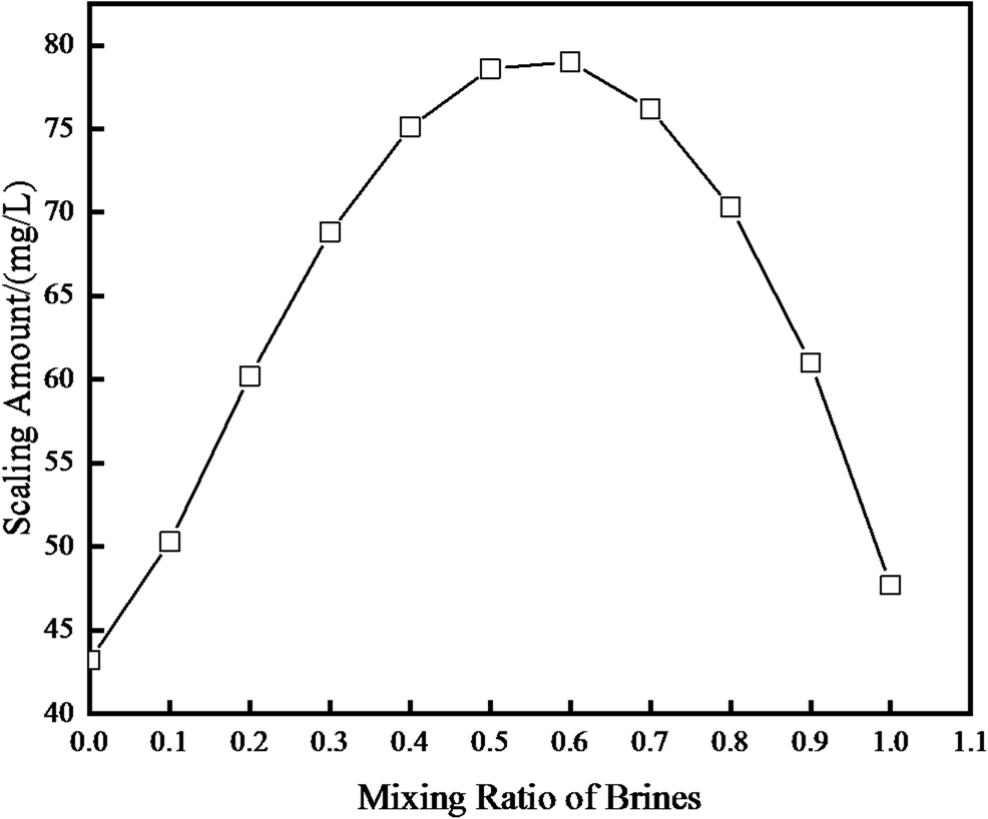
At reservoir circumstances, the prediction results for a mixture of produced water of Yan ‘an Formation and surface water.

As illustrated in Fig. 3, the scaling trend prediction results show that mixing the Yan’an Formation produced water with surface water primarily leads to the formation of calcium carbonate (CaCO₃) scale, with the total scaling amount remaining below 80 mg/L.

This observation directly indicates poor compatibility between the Yan’an Formation produced water and surface water—even though the overall scaling amount is relatively low compared to other water source combinations (e.g., Chang 6 reservoir produced water mixed with Yan’an Formation produced water), the inherent tendency to form CaCO₃ scale still confirms that these two waters are not fully compatible for mixed injection.

#### Compatibility of Chang 6 reservoir produced water and surface water

**Fig. 4:**
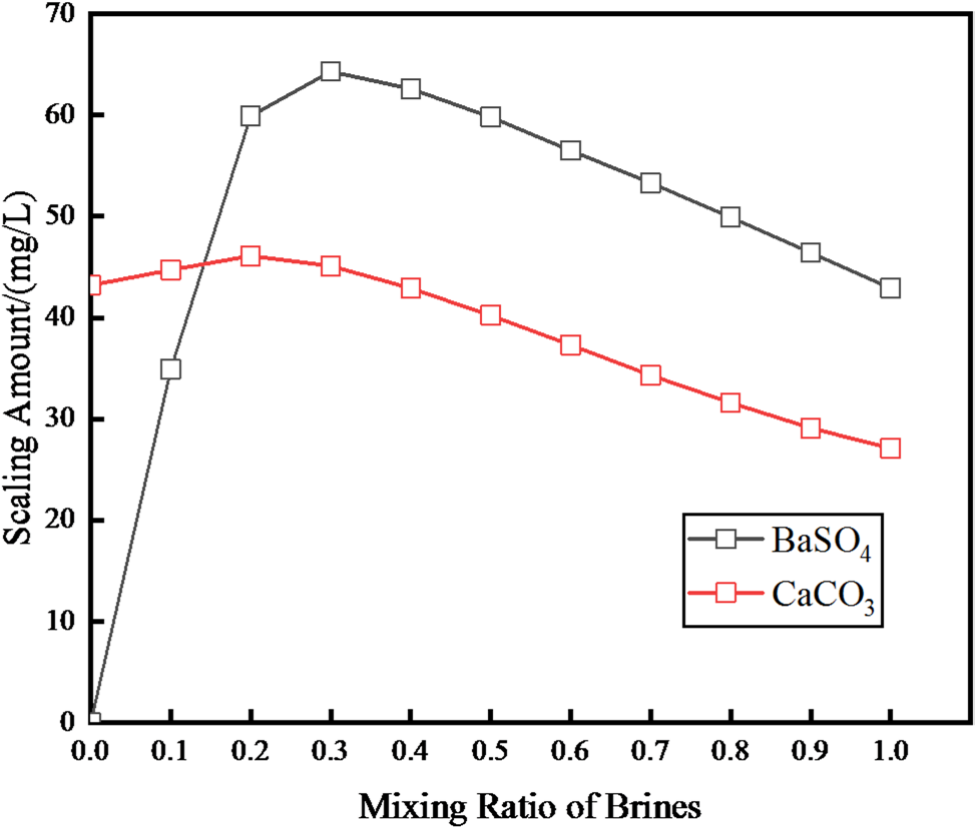
At reservoir circumstances, the prediction results for a mixture of produced water of Chang 6 reservoir and surface water.

When the produced water from the Chang 6 reservoir is mixed with surface water, two types of inorganic scale—calcium carbonate (CaCO₃) and barium sulfate (BaSO₄)—are formed, as illustrated in Fig. 4.

For BaSO₄ scale, its formation quantity rises rapidly as the proportion of Chang 6 reservoir produced water (hereafter referred to as “Chang 6 brine”) increases, reaching a peak when the Chang 6 brine ratio is 0.3, after which it gradually decreases^36,37^. The maximum formation quantity of BaSO₄ scale is less than 70 mg/L. In comparison, the peak formation quantity of CaCO₃ scale is under 50 mg/L throughout all mixing ratios.

These results clearly highlight the poor compatibility between the Chang 6 reservoir produced water and surface water, confirming that their mixture inherently carries a scaling risk.

#### Compatibility of Yan’an Formation and Chang 6 reservoir produced water

In the Shuanghe Oilfield, the Chang 6 reservoir acts as the primary production layer, with several reservoirs from the Yan’an Formation also contributing to production. Produced waters from both these layers are collected at a nearby wastewater treatment station, where crude oil and suspended solids are removed prior to the water being reinjected into the Chang 6 reservoir. However, given the substantial differences in ion composition and water type between the produced water from the Chang 6 reservoir and that from the Yan’an Formation, investigating the compatibility of these two water sources is of critical importance.

**Fig. 5:**
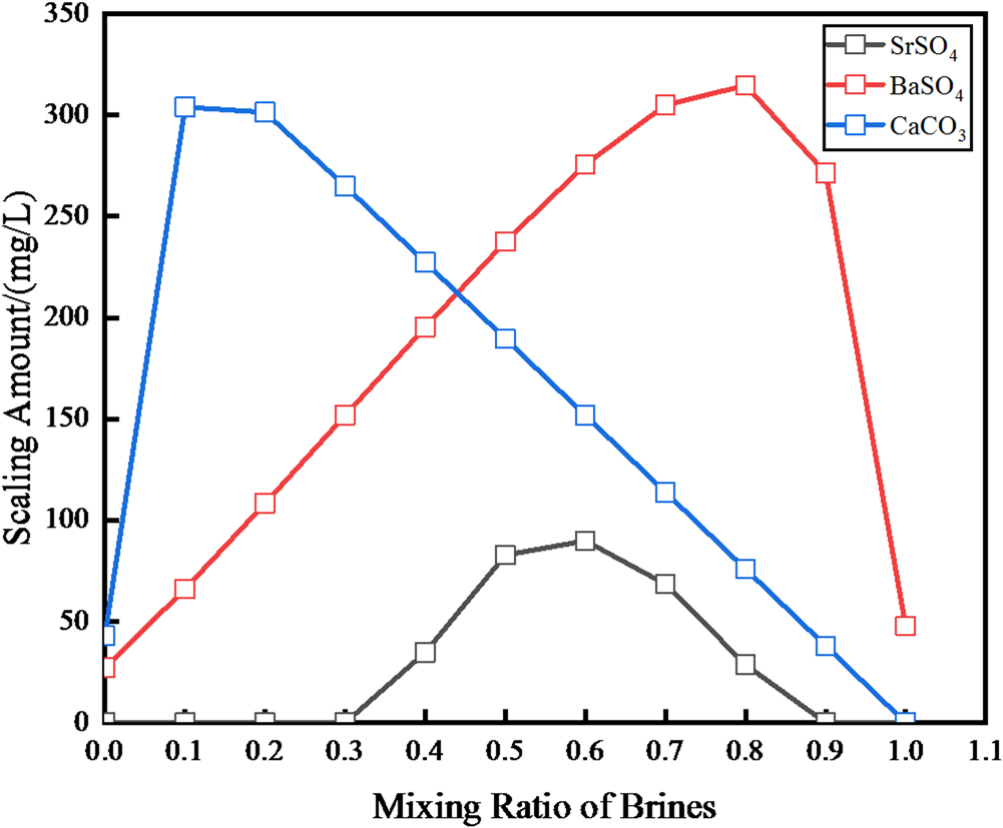
At reservoir circumstances, the prediction results for a mixture of produced water of Chang 6 reservoir with that of Yan’an Formation.

As shown in Fig. 5, the results indicate that mixing these two produced waters leads to the formation of three types of scale: calcium carbonate (CaCO₃), strontium sulfate (SrSO₄), and barium sulfate (BaSO₄). Notably, across all Yan’an Formation brine mixing ratios ranging from 0.1 to 0.9, the formation amounts of CaCO₃ and BaSO₄ both exceed 100 mg/L. For CaCO₃ scale, the maximum formation amount (up to 314.6 mg/L) occurs at a Yan’an brine ratio of 0.2; in contrast, BaSO₄ scale reaches its peak (303.9 mg/L) at a Yan’an brine ratio of 0.9. SrSO₄ scale forms only when the Yan’an brine ratio is between 0.1 and 0.7, and its formation amount remains below 100 mg/L throughout this range.

These findings emphasize the poor compatibility between the two produced water sources, demonstrating that mixing them at any ratio will result in inorganic salt scaling. This inherent incompatibility is a key driver of the high water injection pressures observed in the field, which in turn highlights the urgency of addressing scaling issues to ensure the efficiency of waterflood development operations^32^.

#### Validation via actual scale sample analysis

**Fig. 6:**
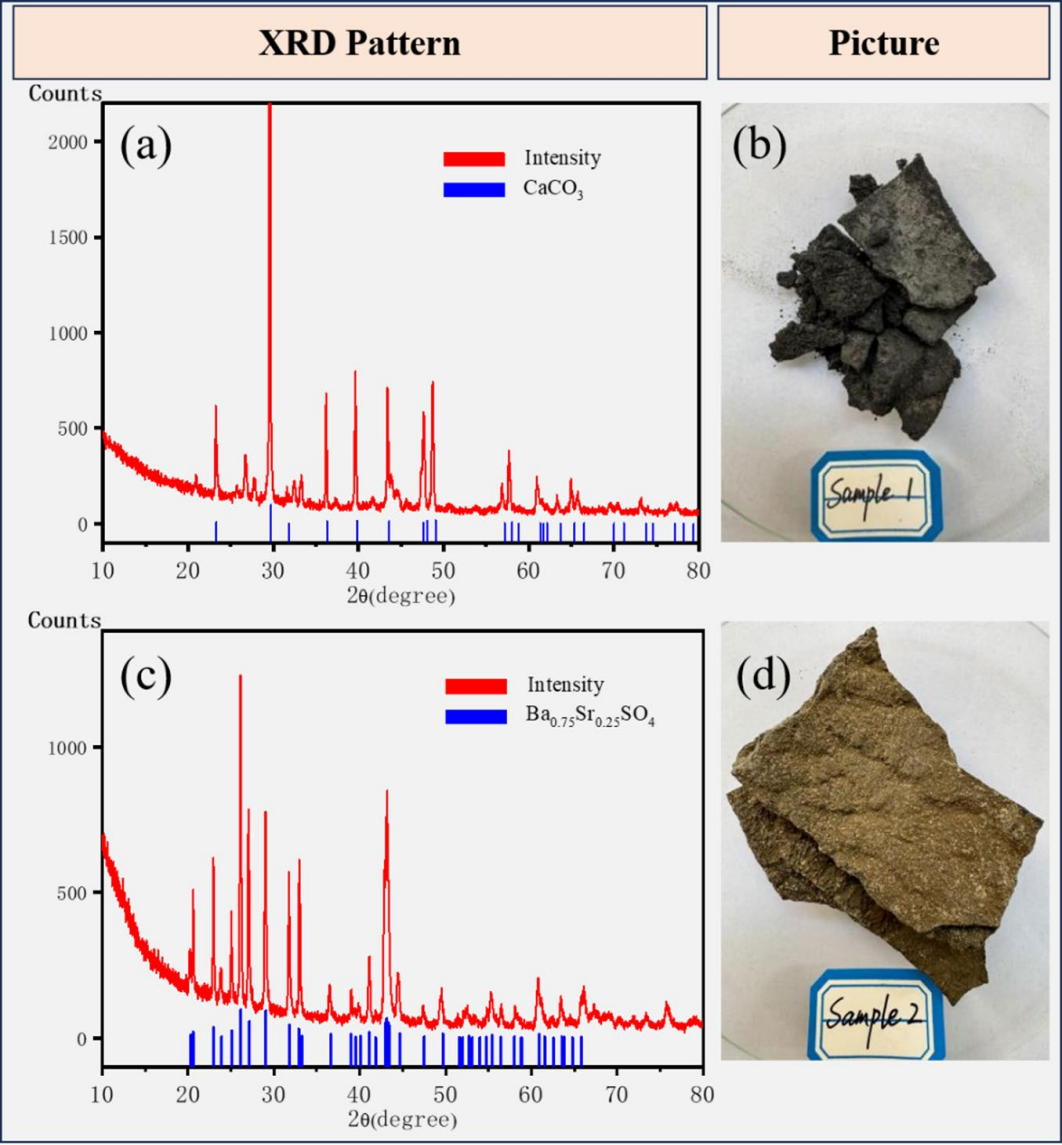
(**a**) XRD pattern of scale sample 1; (**b**) Picture of sample 1; (**c**) XRD pattern of scale sample 2; (**d**) Picture of sample 2.

The collected scale samples were first dried to remove moisture, then analyzed using X-ray diffraction (XRD) to determine their mineral composition. As illustrated in Fig. 6, the XRD patterns reveal distinct compositional characteristics of the two scale samples: Scale Sample 1 is predominantly composed of calcium carbonate (CaCO₃), while Scale Sample 2 is identified as barium-strontium sulfate scale (a mixed phase of BaSO₄ and SrSO₄)^38,39^.

Crucially, the compositional results of the actual scale samples are in good agreement with both the theoretical scaling trend predictions (from ScaleChem software) and the findings of the laboratory compatibility experiments. This consistency validates the reliability of our earlier analysis of scaling types and mechanisms, forming a closed loop between theoretical prediction, laboratory testing, and on-site verification.

The XRD analysis confirms that the field scale samples are predominantly composed of calcite (CaCO₃) and a barite-celestine solid solution (Ba, Sr)SO₄. This composition aligns closely with the ScaleChem predictions, which identified CaCO₃ and BaSO₄/SrSO₄ as the dominant scale types under the key mixing ratios relevant to field conditions (e.g., mixtures of Chang 6 and Yan’an Formation produced waters). While a direct quantitative comparison of predicted versus collected mass is challenging due to the complexity of field deposition environments, the consistent identification of the primary scale types provides strong validation for the applicability of the ScaleChem model to the specific geochemical system of the Shuanghe oil area.

### Key factors influencing scaling behavior

#### Effects of temperature and pH on scaling amount

In the Shuanghe Oilfield, predictions regarding scaling from mixing two distinct types of produced water—namely, from the Yan’an Formation and the Chang 6 reservoir—indicate that mixing these waters will lead to the precipitation of calcium carbonate (CaCO₃), barium sulfate (BaSO₄), and strontium sulfate (SrSO₄). It is well established that the formation of inorganic insoluble salts, particularly CaCO₃, is significantly affected by water temperature and pH. Therefore, experiments were designed to investigate how temperature and pH influence the maximum scaling amount in the mixed produced waters.

The effect of temperature on scaling was tested under atmospheric pressure, with mixed water ratios selected to induce maximum scaling for each scale type: a Chang 6 brine ratio of 0.1 for peak BaSO₄ formation, 0.6 for peak SrSO₄ formation, and 0.8 for peak CaCO₃ formation. For context, the produced water from the Yan’an Formation has a natural pH of 8.5, while that from the Chang 6 reservoir has a natural pH of 6.2. The results of this temperature-dependent assessment are summarized in Table 5.

Separately, the influence of pH on maximum scaling amount was evaluated under normal pressure and a constant temperature of 50 °C (consistent with reservoir temperature conditions). The mixed water ratios used here were identical to those in the temperature tests; the only variable adjusted was the pH of the mixed water. The findings from this pH-focused evaluation are presented in Table 6.

As shown in Tables 5 and 6, increases in both water temperature and pH lead to a greater amount of CaCO₃ scaling. This trend can be explained by two key mechanisms: first, the solubility of CaCO₃ decreases as temperature rises, promoting precipitation; second, a higher pH drives the conversion of bicarbonate ions (HCO₃⁻) to carbonate ions (CO₃²⁻), which then react with free calcium ions (Ca²⁺) in the water to form insoluble CaCO₃. In contrast, temperature has a minimal impact on BaSO₄ and SrSO₄ scaling: a 10 °C increase in temperature only increases their solubility by approximately 4–5 mg/L. Additionally, pH has little effect on the formation of these two sulfate scales—a observation that underscores the stability of BaSO₄ and SrSO₄ as strong acid-strong base salts, whose solubility is largely unaffected by changes in aqueous acidity^40,41^.

The promoting effect of increasing temperature on CaCO₃ precipitation is consistent with observations in CO₂-brine systems, where higher temperatures generally favor the carbonate mineralization reaction, a key mechanism for permanent CO₂ trapping. This parallel underscores the common thermodynamic drivers for carbonate scale formation in both engineered waterflood and natural geochemical storage contexts.

**Table 5:** The effect of temperature on the amount of scaling.

Temperature/℃	20	30	40	50	60
BaSO_4_(brine of Chang 6:0.1) mg/L	317.5	313.2	308.6	303.9	299.0
SrSO_4_(brine of Chang6:0.6) mg/L	73.2	64.6	62.6	61.4	62.0
CaCO_3_(brine of Chang6: 0.8) mg/L	214.7	247.1	280.5	314.6	348.7

**Table 6:** The effect of pH value on the amount of scaling.

pH	6.0	6.5	7.0	7.5	8.0	8.5
BaSO_4_ (brine of Chang 6:0.1)mg/L	303.8	303.8	303.8	303.8	303.8	303.8
SrSO_4_ (brine of Chang 6:0.6) mg/L	0	25.70	37.02	55.14	73.20	73.20
CaCO_3_ (brine of Chang6:0.8)mg/L	0	54.35	152.0	289.0	306.6	326.8

#### Mechanism analysis of scaling

The scaling trend prediction results for the injected water in the Shuanghe Oilfield showed that when produced water is mixed with surface water, the total scale formation amount is less than 100 mg/L, with calcium carbonate (CaCO₃) scale being the dominant type. In contrast, the compatibility between produced waters from different formations is extremely poor: mixing any two types of produced water inevitably results in the formation of three types of scale, namely calcium carbonate, barium sulfate (BaSO₄), and strontium sulfate (SrSO₄).

This scaling phenomenon is closely related to the ionic composition of the produced water and surface water in the oilfield. Specifically, the produced water from the Chang 6 reservoir has high concentrations of calcium ions (Ca²⁺), strontium ions (Sr²⁺), and barium ions (Ba²⁺); the produced water from the Yan’an Formation is rich in scale-forming anions, including bicarbonate ions (HCO₃⁻), sulfate ions (SO₄²⁻), and carbonate ions (CO₃²⁻); while the surface water contains small amounts of HCO₃⁻, SO₄²⁻, and CO₃²⁻.

According to the solubility product principle, scale formation occurs when the activity product of the cation and anion of an insoluble salt in water exceeds the salt’s solubility product constant (Ksp). … For the water systems studied herein, the formation of CaCO₃ scale—one of the main scale types—is essentially determined by the activity product of Ca²⁺ and CO₃²⁻ in the water.

The relevant reaction equations are as follows:

2 $${\mathrm{Ca}}^{{2 + }} + {\mathrm{CO}}_{3}^{{2 - }} \to {\mathrm{CaCO}}_{3} \downarrow$$

3 $${\mathrm{Ca}}^{{{\mathrm{2}} + }} + {\mathrm{2HCO}}_{{\mathrm{3}}} ^{ - } \to {\mathrm{CaCO}}_{{\mathrm{3}}} \downarrow + {\mathrm{H}}_{{\mathrm{2}}} {\mathrm{O}} + {\mathrm{CO}}_{{\mathrm{2}}} \uparrow$$

When : [Ca^2+^] × [CO_3_^2−^] > Ksp _CaCO3_, there will be a calcium carbonate scaling trend.

Similarly, [Ba^2+^] × [SO_4_^2−^] > Ksp _BaSO4_, [Sr^2+^] × [SO_4_^2−^] > Ksp SrSO_4_, there is a tendency to produce strontium sulfate and barium sulfate scale, the reaction equation is as follows:

4 $${\mathrm{Sr}}^{{{\mathrm{2}} + }} + {\mathrm{SO}}_{{\mathrm{4}}}^{{{\mathrm{2}} - }} \to {\mathrm{SrSO}}_{{\mathrm{4}}} \downarrow$$

5 $${\mathrm{Ba}}^{{{\mathrm{2}} + }} + {\mathrm{SO}}_{{\mathrm{4}}}^{{{\mathrm{2}} - }} \to {\mathrm{BaSO}}_{{\mathrm{4}}} \downarrow$$

The predominance of calcium carbonate (CaCO₃) scale over magnesium carbonate (MgCO₃) and strontium carbonate (SrCO₃) scales can be attributed to the combined effects of their solubility product constants (Ksp) and the specific ion concentrations in the injected water. For sulfate scales, the Ksp values follow a clear order: Ksp (BaSO₄) < Ksp (SrSO₄) < Ksp (CaSO₄). Given the stable concentrations of SO₄²⁻, Ca²⁺, Sr²⁺, and Ba²⁺ in the mixed water, BaSO₄ precipitates first due to its lowest Ksp, followed by SrSO₄^42–44^.

For sulfate scales, the Ksp values follow a clear order: Ksp (BaSO₄) < Ksp (SrSO₄) < Ksp (CaSO₄). Given the stable concentrations of SO₄²⁻, Ca²⁺, Sr²⁺, and Ba²⁺ in the mixed water, BaSO₄ precipitates first due to its lowest Ksp, followed by SrSO₄. In contrast, CaSO₄ has a much higher Ksp, meaning its ion activity product in the injected water never reaches the threshold for precipitation—thus, CaSO₄ scale does not form in the system.

In summary, when surface water is injected into either the Yan’an Formation reservoir or the Chang 6 reservoir, only a small amount of CaCO₃ scale is generated. However, the produced water from the Yan’an Formation is highly incompatible with that from the Chang 6 reservoir. Currently, the Shuanghe Oilfield adopts a cost-effective approach of mixing and treating these formation waters before reinjection into the Chang 6 reservoir. Unfortunately, this untreated mixed produced water (prior to the optimized process) leads to severe scaling on injection pipelines and equipment. Therefore, targeted anti-scaling measures are essential to mitigate this issue.

### Optimization of wastewater treatment process and its effect

#### Adjustment of sewage treatment process

Common scale prevention methods in oilfields include chemical scale inhibition, physical scale inhibition, and process-based scale inhibition. Based on the characteristics of reinjection water and reservoirs in the Shuanghe Oilfield, this study adopted the process-based scale inhibition method.

Our analysis emphasizes that controlling the concentrations of sulfate ions (SO₄²⁻) and carbonate ions (CO₃²⁻) in injected water is critical to improving the compatibility of mixed water injection. To this end, a pre-mixing tank was added to the existing wastewater treatment process, positioned between the oil-water separator and the sewage treatment system. Additionally, to effectively prevent calcium carbonate (CaCO₃) scale formation, the pH value of the mixed water was monitored online, and acid regulators were used to adjust it to 6.0–6.5—a range consistent with the pH of the Chang 6 reservoir’s in-situ water.

This two-pronged strategy of pre-mixing for sulfate removal and pH adjustment constitutes an engineered approach to manage fluid compatibility. It is conceptually analogous to techniques like water-alternating-gas (WAG) or sequenced brine injection used in CO₂ storage and enhanced oil recovery, which are designed to actively control fluid-fluid and fluid-rock interactions to achieve desired outcomes (e.g., improved trapping, reduced impairment).

In the Shuanghe Oilfield, the core to enhancing injected water compatibility lies in controlling sulfate ion concentrations. Specifically, the produced water from the Chang 6 reservoir (which contains barium ions (Ba²⁺) and strontium ions (Sr²⁺)) undergoes full reaction with the produced water from the Yan 9 and Yan 10 reservoirs (which contains sulfate ions) in the pre-mixing tank. This reaction generates barium-strontium sulfate scale precipitates, which are subsequently removed from the bottom of the tank. It is worth noting that the Chang 6 layer is the primary production layer of the oilfield, and its produced water volume is significantly larger than that of the Yan 9 and Yan 10 reservoirs.

**Fig. 7:**
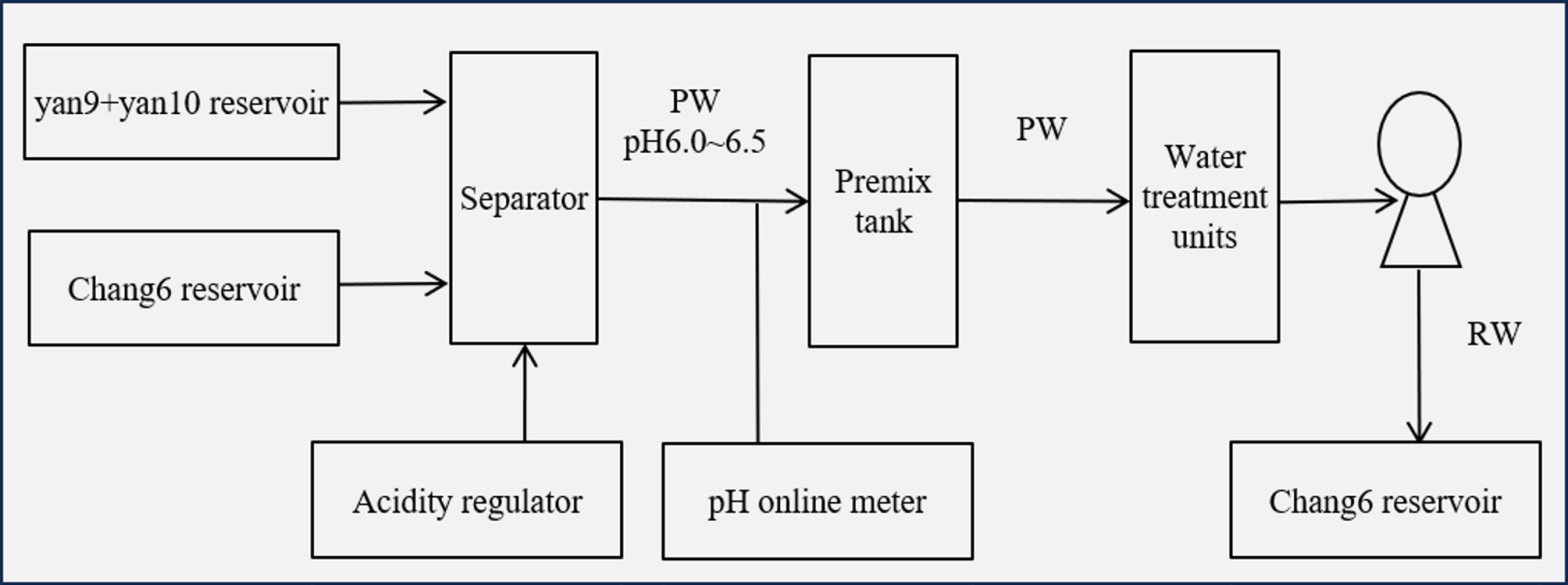
Schematic diagram of wastewater treatment process adjustment; PW: produced water, IW: reinjection water.

Following the adjustment of the water treatment process, three months of continuous monitoring showed that the sulfate ion content in the reinjected water remained below 50 mg/L, and the pH of the reinjected water was stabilized between 6.0 and 6.5. This effectively suppressed the formation of calcium carbonate scale. The optimized produced water treatment process is illustrated in Fig. 7.

Environmental and Practical Considerations. The optimized process effectively prevents scale formation downhole, but it transfers the precipitation of barium and strontium sulfates to the surface pre-mixing tank. This generates a solid sludge that requires proper handling and disposal in accordance with local environmental regulations. However, this approach eliminates the need for continuous injection of chemical scale inhibitors into the reservoir, thereby avoiding potential long-term formation damage and groundwater contamination risks associated with such chemicals. The trade-off between managing a concentrated solid waste stream at the surface versus dispersing chemicals underground represents a shift towards a more controlled and potentially sustainable waste management strategy for this specific scaling problem^45^.

#### Effect of optimized process on core damage rate

To assess and validate the effectiveness of the optimized water treatment process and scale inhibitor in mitigating scale formation, displacement experiments were conducted using reservoir cores collected from the target water injection block. The results, summarized in Table 7, provide valuable insights into how different treatments influence the core flow damage rate under actual reservoir conditions.

When untreated mixed water was used as the displacing medium in the displacement experiments, the core flow damage rate exceeded 45%. This high damage rate is attributed to extensive scaling and subsequent pore blockage in the cores, which are driven by chemical reactions between incompatible ions in the mixed water. In contrast, when the mixed-layer water was subjected to pre-mixing and scale inhibitor treatment prior to being used as the displacing medium, the core flow damage rate was significantly reduced to below 20%. This marked reduction clearly demonstrates that the proposed treatment process is effective in suppressing scale formation and alleviating core blockage^46^.

The observed improvement underscores that the water treatment process—incorporating pre-mixing of produced water from different layers and pH adjustment to 6.0–6.5—can effectively resolve the issue of scale-induced blockage during water injection in low-permeability reservoirs. This approach offers a practical and efficient solution for managing mixed water injection in scenarios involving multiple types of produced water, thereby enhancing the overall efficiency and sustainability of waterflood development operations in the oilfield.

**Table 7:** Optimization of waterflood treatment in Shuanghe oilfield.

displacing medium	Core	Displacement pressure difference MPa	Temperature ℃	Effective permeability 10^−3^µm^2^	Damage rate %
Untreated mixed water	shuang12-2	2.0	50	1.15	45.8
Mix water after treatment	shuang12-4	2.0	50	1.56	17.6
Untreated mixed water	shuang12-5	2.3	50	0.97	49.5
Mix water after treatment	shuang12-6	2.5	50	0.88	19.3

### Discussion and implications

The treatment strategy developed in this work is specifically designed to address the scaling challenge in the Shuanghe oil area, which is characterized by the incompatibility between barium/strontium-rich water and sulfate-rich water. Therefore, its direct application to other oilfields requires a prior diagnostic analysis to identify the specific incompatible ion pairs (e.g., Ca²⁺/HCO₃⁻ for carbonate scale, Fe²⁺/HS⁻ for sulfide scale) and scaling mechanisms prevalent there.

The broader significance of this study lies in the integrated workflow it demonstrates: (1) comprehensive compositional analysis and compatibility assessment of all potential injection water sources; (2) utilization of reliable scaling prediction software (ScaleChem) to quantify scaling tendencies and identify dominant scale types under various mixing scenarios; (3) experimental investigation of key controlling factors (e.g., pH, temperature); and (4) the design and validation of a targeted, process-based inhibition method tailored to the identified key factors. This systematic workflow provides a replicable template for diagnosing and addressing mixed-water scaling issues in other oilfields, even when the dominant scaling ions differ from those in Shuanghe.

## Conclusion

1. This study, focused on the Shuanghe oil area, confirms that the extreme difference in ionic composition between the Chang 6 reservoir produced water (high in Sr²⁺, 408.0 mg/L, and Ba²⁺, 225.9 mg/L) and the Yan’an Formation produced water (high in SO₄²⁻, > 1100 mg/L) is the primary cause of severe scaling and associated reservoir damage during mixed injection in this low-permeability oilfield, resulting in elevated injection pressure (as injectivity declines) and reduced waterflood efficiency.
2. The scale trend prediction method employing ScaleChem software proved effective for evaluating scaling tendencies under the studied conditions. The implemented pre-mixing treatment successfully addressed the incompatibility for barium-strontium sulfate scale, yielding mixed water with good compatibility. This compatibility was shown to be stable under simulated reservoir temperature and pressure, as well as upon dilution with in-situ formation water.
3. For the Shuanghe oil area, controlling the sulfate ion (SO₄²⁻) concentration and the pH of the injected water are identified as critical. The combined treatment process—ground pre-mixing followed by pH adjustment to 6.0–6.5—successfully reduced the sulfate ion concentration to below 50 mg/L. This process-based approach effectively mitigated scaling, resulting in a core damage rate of only 19.3% after 10 pore volumes (PV) of injection in laboratory experiments. Therefore, this strategy presents a viable solution to the scaling problem in this specific oilfield. Future work should focus on evaluating its long-term economic feasibility and exploring its potential adaptation to other oilfields with analogous scaling mechanisms but differing water chemistries.
4. Finally, this study demonstrates that effective scale management in waterflood projects requires a holistic understanding of the system’s specific water chemistry, mineralogy, and the dynamic interactions between injected and formation waters. The integrated workflow presented here—combining predictive modeling, experimental validation, and process optimization—provides a systematic approach for diagnosing and mitigating scaling issues in oilfields with complex water sources.

## Supplementary Information

Below is the link to the electronic supplementary material.

Supplementary Material 1

## Data Availability

The datasets used and/or analyzed during the current study available from the corresponding author on reason-able request.
