# Supplementary material for: The analysis and control of scale accumulation for mixed layer injection of water for the Shuanghe oil area in Yanchang Oilfield
Source: Sci Rep. 2026 Apr 9;16:15733. doi: 10.1038/s41598-026-47479-6 (PMC13190844; doi:10.1038/s41598-026-47479-6)
Supplement: Supplementary file 1 — Supplementary Material 1 [file 41598_2026_47479_MOESM1_ESM.pdf]

Data availability

The datasets used and/or analyzed during the current study available from the corresponding author on reason-able request.
